# HCN4 subunit expression in fast-spiking interneurons of the rat spinal cord and hippocampus

**DOI:** 10.1016/j.neuroscience.2013.01.028

**Published:** 2013-05-01

**Authors:** D.I. Hughes, K.A. Boyle, C.M. Kinnon, C. Bilsland, J.A. Quayle, R.J. Callister, B.A. Graham

**Affiliations:** a Spinal Cord Research Group, Institute of Neuroscience and Psychology, College of Medical, Veterinary and Life Sciences, University of Glasgow, Glasgow G12 8QQ, United Kingdom; b School of Biomedical Sciences and Pharmacy, The University of Newcastle, Callaghan, NSW 2308, Australia; c Hunter Medical Research Institute (HMRI), Rankin Park, Newcastle, NSW 2287, Australia

**Keywords:** AP, action potential, CB, calbindin, ChAT, choline acetyltransferase, HCN, hyperpolarisation-activated cyclic nucleotide-gated, IaINs, group Ia inhibitory interneurons, PV, parvalbumin, PKCγ, protein kinase C gamma, RCs, Renshaw cells, TSA, tyramide signal amplification, HCN channels, spinal cord, hippocampus, interneurons, channelopathy

## Abstract

Hyperpolarisation-activated (*I_h_*) currents are considered important for dendritic integration, synaptic transmission, setting membrane potential and rhythmic action potential (AP) discharge in neurons of the central nervous system. Hyperpolarisation-activated cyclic nucleotide-gated (HCN) channels underlie these currents and are composed of homo- and hetero-tetramers of HCN channel subunits (HCN1–4), which confer distinct biophysical properties on the channel. Despite understanding the structure–function relationships of HCN channels with different subunit stoichiometry, our knowledge of their expression in defined neuronal populations remains limited. Recently, we have shown that HCN subunit expression is a feature of a specific population of dorsal horn interneurons that exhibit high-frequency AP discharge. Here we expand on this observation and use neuroanatomical markers to first identify well-characterised neuronal populations in the lumbar spinal cord and hippocampus and subsequently determine whether HCN4 expression correlates with high-frequency AP discharge in these populations. In the spinal cord, HCN4 is expressed in several putative inhibitory interneuron populations including parvalbumin (PV)-expressing islet cells (84.1%; SD: ±2.87), in addition to all putative Renshaw cells and Ia inhibitory interneurons. Similarly, virtually all PV-expressing cells in the hippocampal CA1 subfield (93.5%; ±3.40) and the dentate gyrus (90.9%; ±6.38) also express HCN4. This HCN4 expression profile in inhibitory interneurons mirrors both the prevalence of *I_h_* sub-threshold currents and high-frequency AP discharge. Our findings indicate that HCN4 subunits are expressed in several populations of spinal and hippocampal interneurons, which are known to express both *I_h_* sub-threshold currents and exhibit high-frequency AP discharge. As HCN channel function plays a critical role in pain perception, learning and memory, and sleep as well as the pathogenesis of several neurological diseases, these findings provide important insights into the identity and neurochemical status of cells that could underlie such conditions.

## Highlights

► HCN4 channel expression in selected fast-spiking spinal interneuron populations. ► HCN4 channels expressed in most fast-spiking hippocampal interneurons. ► HCN4 channels rarely expressed in regular- or gap-firing interneurons. ► HCN channelopathies now implicated in neurodegenerative diseases and chronic pain.

## Introduction

Neurons in the central nervous system (CNS) exhibit diverse physiological, morphological and neurochemical properties and these features have been used as defining criteria to help identify functionally distinct populations. For example, the discharge responses of rodent spinal dorsal horn neurons to depolarising current injection is highly variable and can be described as tonic firing, initial bursting, delayed firing or single spiking (Thomson et al., 1989; Grudt and Perl, 2002). Importantly, these action potential (AP) discharge patterns appear to be related to both neurotransmitter phenotype and morphology (Grudt and Perl, 2002; Graham et al., 2004, 2008; Yasaka et al., 2007, 2010). Islet cells, for example, form a morphologically distinct population of inhibitory interneurons in the spinal dorsal horn that typically discharge APs at very high frequencies and express *I_h_* currents (Grudt and Perl, 2002; Yasaka et al., 2007, 2010). Hyperpolarisation-activated cyclic nucleotide-gated (HCN) channels are known to play important roles in both establishing *I_h_* currents and contributing to high-frequency AP discharge patterns in a range of tissues including central and peripheral neurons, cardiac myocytes and taste cells (Moosmang et al., 2001; Stevens et al., 2001; Notomi and Shigemoto, 2004; Baruscotti et al., 2005). Four genes are known to encode for HCN channel proteins and these homologous HCN channel subunits (HCN1–4) can assemble as homomeric or heteromeric tetramer complexes to form channels with differing kinetics and voltage activation profiles (Wahl-Schott and Biel, 2009). Importantly, these properties allow HCN channels to play a critical role in setting resting membrane potential, regulating repetitive AP discharge, and shaping dendritic processing in neuron populations involved in learning, memory and pain (Lüthi and McCormick, 1998; Bennett et al., 2000; Dudman and Nolan, 2009; Oswald et al., 2009). More recently, HCN channelopathies have been implicated in the pathophysiology of neurological diseases such as epilepsy and have therefore been proposed as potential targets for future drug therapies (Lewis and Chetkovich, 2011; Reid et al., 2012).

Several studies have surveyed HCN channel subunit expression in the rodent CNS and show that HCN1 and HCN2 are the most widely distributed forms (Moosmang et al., 1999; Santoro et al., 2000; Notomi and Shigemoto, 2004). As a consequence, studies on *I_h_* currents and pacemaker activity in neurons have focused principally on HCN1 and HCN2 subunits despite both HCN3 and HCN4 also being critical for establishing rhythmic firing in certain neuronal populations (Cho et al., 2009; Ying et al., 2011). Furthermore, recent studies show that these subunits are more prevalent in the spinal cord and hippocampus than previously thought, and are expressed in cells that exhibit high-frequency AP discharge (Brewster et al., 2007; Hughes et al., 2012). Despite the documented differences in the biophysical properties of HCN channels in expression systems, little is known about the expression and stoichiometry of these channels in the intact nervous system or how expression patterns are likely to contribute to neuron function*.* As HCN channelopathies are now being implicated in a number of neurological diseases including epilepsy and chronic pain (Chaplan et al., 2003; Lewis and Chetkovich, 2011), we focus on HCN4 expression in defined subpopulations of neurons in the spinal cord and hippocampus. This strategy will begin to address the paucity of information available on the distribution of this and other HCN subunits in these clinically relevant regions.

HCN channel subunit expression is likely to be an important determinant of membrane properties in CNS neurons and we propose that HCN4 expression in these selected inhibitory interneuron populations is linked to high-frequency AP discharge and the presence of *I_h_* currents (Southan et al., 2000; Dudman and Nolan, 2009). To test this hypothesis, we have used the neurochemical profiles of spinal and hippocampal neurons to first identify populations reported to exhibit either high-frequency AP discharge (e.g. islet cells, Renshaw cells (RCs), Ia inhibitory interneurons, basket cells and oriens-lacunosum moleculare cells) or transient/regular-spiking discharge patterns (motor neurons, pyramidal cells, granule cells) and then determine whether they express immunolabelling for the HCN4 channel subunit.

## Experimental procedures

All experiments were approved by the University’s Ethical Review Process Applications Panel and were performed in accordance with the European Community directive 86/609/EC and the United Kingdom Animals (Scientific Procedures) Act 1986.

### Fixation and tissue preparation

A total of nine adult male Wistar rats (220–290 g; Harlan UK Ltd., Bicester, UK) were deeply anaesthetised with pentabarbitone and perfused transcardially with 4% depolymerised formaldehyde. The brain and lumbar spinal cord were removed and post-fixed in the same solution for an additional 2 h. Spinal cord (transverse and parasagittal planes) and hippocampal sections (coronal plane) were cut on a Vibratome (60-μm thick) and subsequently incubated in 50% ethanol for 30 min to enhance antibody penetration.

Free-floating spinal cord and hippocampal sections were incubated in either goat anti-parvalbumin (PV), rabbit anti-calbindin (CB) and mouse anti-HCN4 or goat anti-PV, rabbit anti-PKCγ and mouse anti-HCN4 for 72 h. All of these antibodies are available from commercial sources, are widely used and have been fully characterised (see Table 1 for details). HCN4 labelling was visualised using a tyramide signal amplification (TSA) step, whereas immunolabelling for calcium-binding proteins and PKCγ was visualised using species-specific secondary antibodies conjugated to either Alexa 488 or Cy5. Sections were incubated overnight in fluorescent-labelled secondary antibodies and biotinylated donkey anti-mouse. They were subsequently incubated for 3 h in Avidin conjugated to horseradish peroxidase, before carrying out a TSA reaction using a tetramethylrhodamine kit (PerkinElmer Life Sciences, Boston, MA, USA) in accordance with the manufacturer’s instructions.

To examine the expression of HCN4 in spinal motor neurons, we first incubated spinal cord sections in mouse anti-HCN4 prior to revealing immunolabelling using the TSA method as described above. These sections were then incubated in a cocktail of mouse anti-gephyrin (7a) and goat anti-choline acetyltransferase (ChAT) before detecting immunolabelling for these markers using secondary antibodies conjugated to Alexa 488 and Cy5 respectively. All primary and secondary antibody cocktails were made up in 0.3 M phosphate-buffered saline with 0.3% Triton X-100. Sections were incubated in primary antibodies for 72 h and in secondary antibodies for 12–18 h.

### Neurochemical identification of functionally discrete spinal and hippocampal populations

We have used combinations of different neurochemical markers to determine whether functionally defined neuronal populations in the spinal cord and hippocampus express the HCN4 channel subunit (see Table 2). For example, in the spinal dorsal horn, HCN4-immunolabelling is profuse in lamina II. PV-expressing cells are a prominent group of lamina II inhibitory interneurons and their morphology resembles those of islet cells (Antal et al., 1990; Laing et al., 1994). Their dendritic trees are elongated in the rostro-caudal axis (typically extending over 400 μm) but have relatively limited dorso-ventral spread into adjacent laminae. The axons of islet cells arborise extensively within the volume of the dendritic tree but are mostly confined to lamina II (Gobel, 1975; Grudt and Perl, 2002; Yasaka et al., 2010). Islet cells comprise a physiologically homogeneous population of cells, exhibiting tonic-firing AP discharge patterns and *I_h_*-type subthreshold currents and are therefore likely to express HCN channels (Grudt and Perl, 2002; Yasaka et al., 2007, 2010). Cells that express the gamma isoform of protein kinase C (PKCγ) are also common in the ventral part of lamina II (Polgár et al., 1999). Most of these excitatory interneurons express A-type potassium currents but show heterogeneity in both morphology and AP discharge (Polgár et al., 1999; Hu and Gereau, 2011) and are therefore likely to show different patterns of HCN4 immunolabelling compared to PV cells. In the spinal ventral horn, RCs and group Ia inhibitory interneurons (IaINs) also exhibit high-frequency AP discharge (Renshaw, 1946; Eccles et al., 1954; Mentis et al., 2005) and can be identified by co-expression of both CB and PV (Carr et al., 1998; Alvarez et al., 2005; Alvarez and Fyffe, 2007). Finally, α-motor neurons exhibit repetitive AP discharge (Button et al., 2008), are covered in gephyrin puncta (Todd et al., 2006) and can be identified by their expression of ChAT, large soma diameter (>30 μm) and the presence of large cholinergic C-boutons on their cell bodies (Swett et al., 1986; Nagy et al., 1993). In the in *stratum pyramidale* of the hippocampus, PV expression is usually associated with basket cells and axo-axonic cells, both of which typically exhibit high-frequency AP discharge (Kawaguchi et al., 1987; Pawelzik et al., 2002). In contrast, CB immunolabelling in this region identifies bistratified interneurons and sub-populations of pyramidal cells, both of which typically fire with regular-spiking discharge patterns (Ali et al., 1998; Hájos et al., 2004).

### Confocal microscopy and image analysis

Representative sections of the spinal cord and hippocampus from at least three animals for each of the different antibody combinations were scanned on a confocal microscope (Bio Rad Radiance 2100 or Zeiss LSM710, Hemel Hempstead, UK). Image stacks from different regions of the spinal cord and hippocampus were collected using different powered objectives to (i) map the general distribution of HCN4-immunolabelling in spinal cord and hippocampal sections, or (ii) determine the expression of HCN4-immunolabeling in neurochemically defined populations. Montages of tiled images scanned with a ×20 lens were generated using Zen software (Zeiss; Hemel Hempstead, UK) to illustrate patterns of HCN4-immunolabelling. HCN4 expression patterns in neurochemically defined populations was determined from image stacks taken with either ×40, ×60 or ×63 oil immersion lenses (0.3–0.5 μm z-separation) and analysed using Neurolucida for Confocal software (MicroBrightField, Colchester, VT, USA) or Image J. Due to the punctuate nature of HCN4 labelling in some populations, the presence/absence of immunolabelling in individual cells was determined by analysing several optical sections from each cell.

## Results

### Spinal cord

**Fig. 1:**
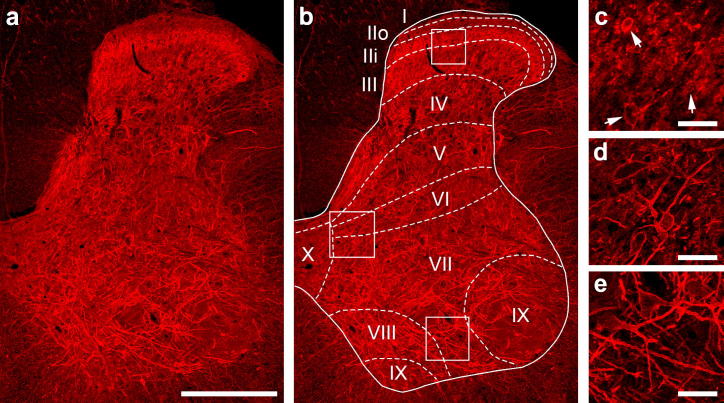
HCN4 expression in the lumbar spinal cord. HCN4 immunolabelling is observed primarily in spinal grey matter, specifically in laminae IIi–VIII (a, b). The intensity of labelling in immunopositive cells varies in dorsal horn laminae IIi and III (c; arrows), but is generally strong in medial regions of the deep dorsal horn (laminae V and VI; d) and ventral horn (lamina VII; e). Immunolabelling is primarily confined to the cell membrane although some cytoplasmic labelling is also evident (b–d). Figures a–e are projected image stacks of 10 optical sections scanned at 1-μm z-separation. Scale bars in a = 250 μm; c = 50 μm, d and e = 100 μm.

HCN4 immunolabelling in the spinal cord is found principally in the spinal grey matter, with some very weak and diffuse staining in the white matter (Fig. 1a, b). In the dorsal horn, immunolabelled cells formed a plexus in lamina II inner (IIi) and in the medial aspect of the deep dorsal horn (laminae V and VI). These cells varied in both size and intensity of HCN4-immunolabelling (Fig. 1c). Although labelling in laminae III and IV was less intense, numerous immunopositive cell bodies and dendrites were still observed. Immunolabelling in laminae I and II outer (IIo) was generally sparse, although occasional cells and stained dendrites were observed. In the ventral horn, immunolabelled cells were located throughout laminae VII and VIII, but were less frequent in laminae IX and X. HCN4-immunolabelling was localised primarily in somatic and dendritic cell membranes of spinal neurons, although some cytoplasmic labelling was also apparent (Fig. 1c–e).

**Fig. 2:**
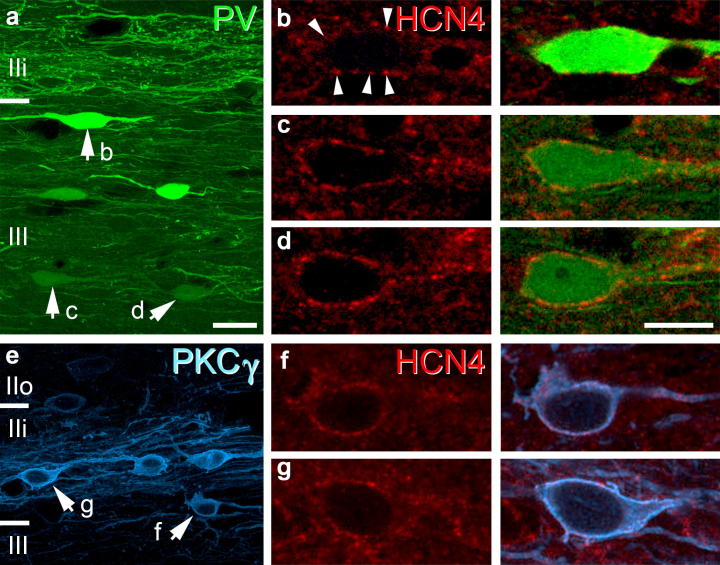
HCN4 expression in PV- and PKCγ-IR dorsal horn neurons – Sagittal sections showing the outer laminae of the dorsal horn. PV-IR cells (green) were mostly found in lamina IIi and III, with PV-expressing axons arborising primarily in lamina IIi (a). Panels b–d show examples of HCN4-expression (red) in the PV cells highlighted in panel a (arrows). HCN4-immunolabelling was present in the majority of PV-IR cells in laminae IIi and III, however the intensity of labelling was variable (b–d). The cell bodies and dendrites of PKCγ-IR cells (blue) in the spinal dorsal horn form a plexus contained principally within lamina IIi (e). HCN4-immunolabelling was found in the cell bodies and dendrites of many of these cells (f, g). Figures are projected image stacks of 6 (Figures a and e) and 2 optical sections (Figures b–d, f and g) scanned at 1-μm z-separation. Scale bars in a = 20 μm; b–d = 10 μm.

**Fig. 3:**
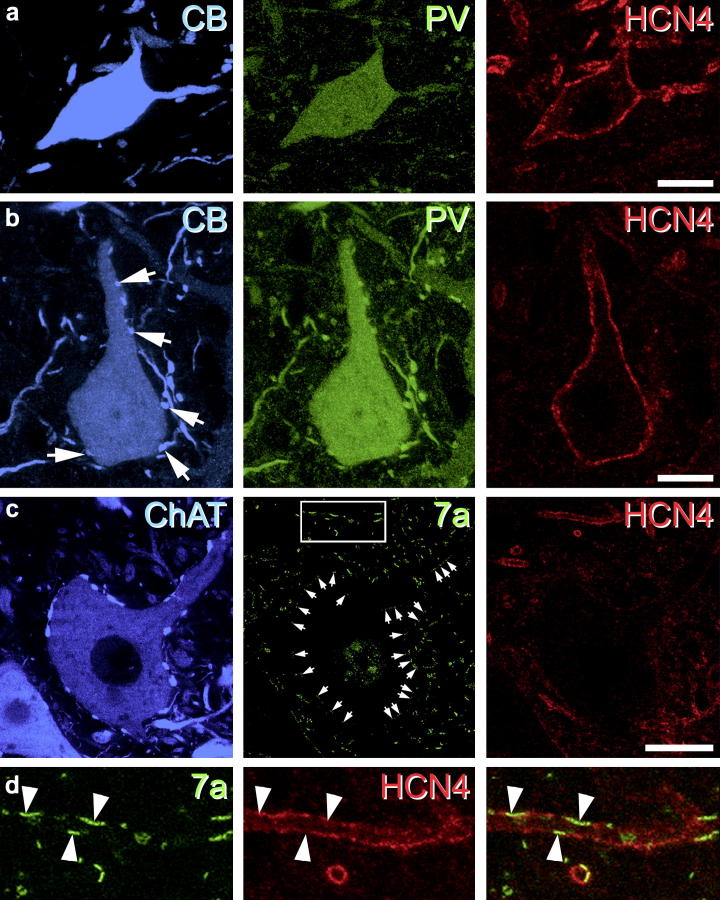
HCN4 expression in ventral horn cells. Renshaw cells (a) are located at the ventral border of lamina VII and IX and were identified by the expression of CB (blue) and PV (green). All Renshaw cells were immunolabelled for HCN4 (red). IaINs (b) were located in lamina VII and at the border between laminae VII and IX. They expressed CB (blue) and were surrounded by a plexus of CB-IR axon terminals (arrowheads) and also contained PV (green). All IaINs were immunolabelled for HCN4 (red). Motor neurons (c) were located in lamina IX and can be identified by their large soma diameter (>30 μm), ChAT immunolabelling (blue), the presence of multiple ChAT-immunopositive C-boutons surrounding their soma and proximal dendrites and presence of gephyrin (7a) expressing puncta in their cell membrane (arrowheads). None of the motor neurons analysed were immunolabelled for HCN4 (red). Note, the specificity of immunolabelling for two mouse primary antibodies (against HCN4 and gephyrin 7a) using a two-step TSA protocol as illustrated by the clear separation of HCN4- (red) and gephyrin-immunolabelling (green) of a nearby unidentified dendrite in the high power insets (d). Figures are projected image stacks of five optical sections scanned at 0.5-μm z-separation. Scale bars in a and b = 10 μm; c = 25 μm.

Most PV-IR cells were found in laminae IIi and III as reported previously (Antal et al., 1990; Laing et al., 1994). A total of 207 PV-IR cells from laminae II and III were analysed, of which 174 expressed HCN4 (mean 84.1%; SD: ±2.87) (Fig. 2). The intensity of HCN4-immunolabelling in these neurons varied, but was generally not as strong as that observed in PV negative HCN4-expressing cells. The distribution of PKCγ-IR cells is more restricted than that of PV-IR cells as they form a dense plexus, which is largely confined to lamina IIi (Hughes et al., 2003). Despite having an overlapping laminar distribution with PV-IR cells, fewer PKCγ-IR cells expressed HCN4. We analysed a total of 291 PKCγ-IR cells, of which 157 were immunolabelled for HCN4 (53.9%; ±3.62). In the ventral horn, PV- and CB-immunolabelled cells were found primarily in laminae VII and VIII, although occasional PV- and CB-IR dendrites and axons projected into lamina IX. Putative RCs, IaINs and motor neurons were identified by their neurochemical profile and laminar distribution (Fig. 3a–c respectively). While all putative RCs and IaINs expressed HCN4-immunolabelling in their cell bodies and dendrites (*n* = 43 and 63, respectively), none of the motor neurons analysed were immunolabelled for HCN4 (*n* = 102). These results are summarised in Fig. 7a.

### Hippocampus

**Fig. 4:**
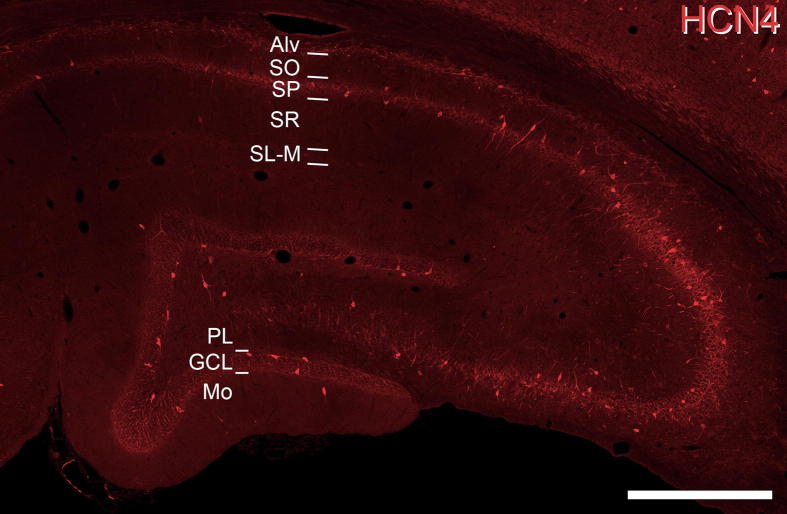
HCN4 expression in the hippocampus. HCN4-immunolabelled cells are primarily located in *stratum pyramidale* (SP), *stratum oriens* (SO) and at the border of *stratum oriens* and the alveus (Alv) in hippocampal subfields CA1 and CA3, but also in the granule and pleomorphic cell layers (GCL and PL, respectively) of the dentate gyrus. This figure is a projected image stack of 12 optical sections scanned at 1 μm z-separation. Scale bar = 500 μm.

**Fig. 5:**
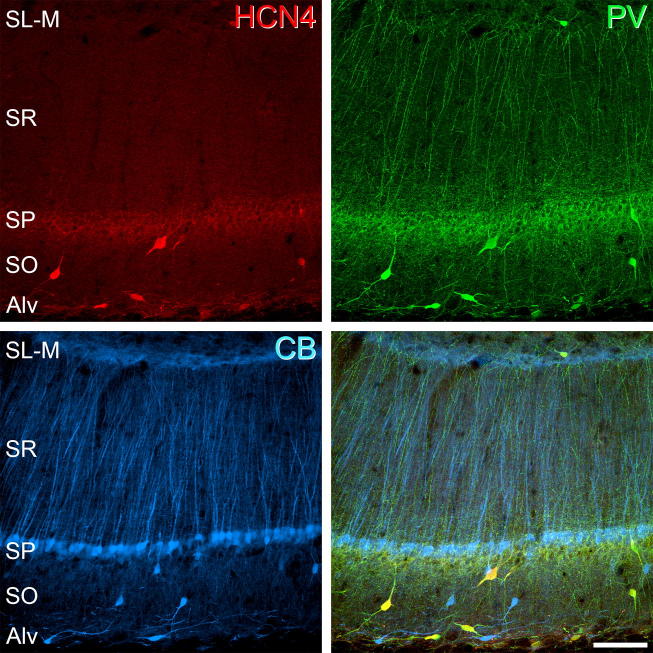
HCN4 expression in the CA1 region of the hippocampus. HCN4-immunolabelled cells (red) were found primarily in *stratum pyramidale* (SP), *stratum oriens* (SO) and at the border of *stratum oriens* and the alveus (Alv). Occasionally cells were observed in *stratum lacunosum-moleculare* (SL-M). Virtually all HCN4-immunolabelled cells expressed PV (green) but not CB (blue). Panels in this figure are projected image stacks of 16 optical sections scanned at 0.5-μm z-separation. Scale bar = 100 μm.

**Fig. 6:**
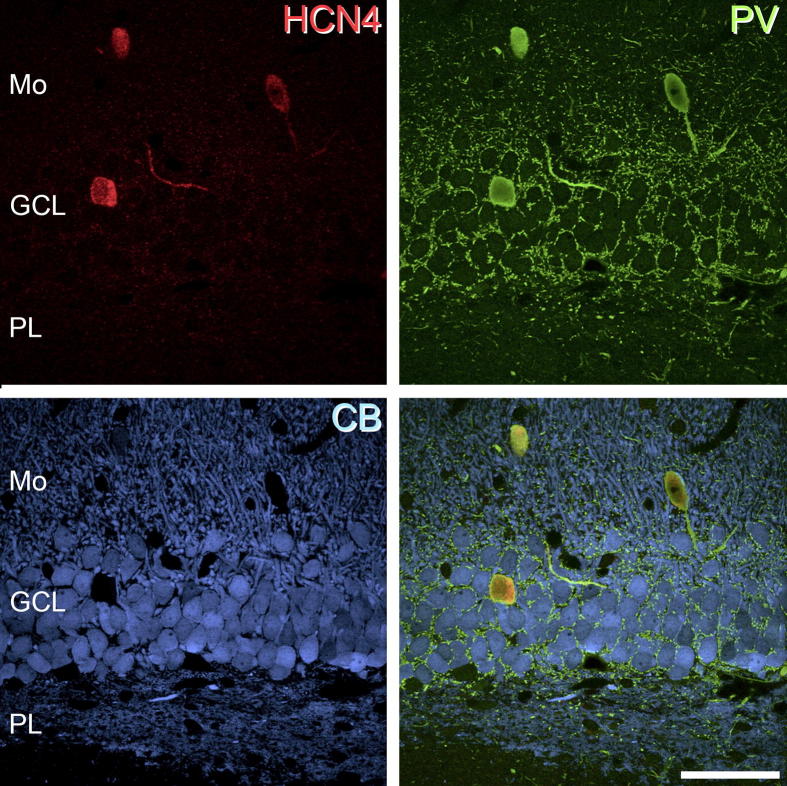
HCN4 expression in the dentate gyrus. HCN4-immunolabelled cells (red) were found primarily in the granule cell layer (GCL) and at the border of the granule and the polymorphic (PL) cell layers. Occasionally cells were observed in the molecular layer (Mo). Virtually all HCN4-immunolabelled cells expressed PV (green) but not CB (blue). The panels in this figure are projected image stacks of eight optical sections scanned at 0.5-μm z-separation. Scale bar = 50 μm.

**Fig. 7:**
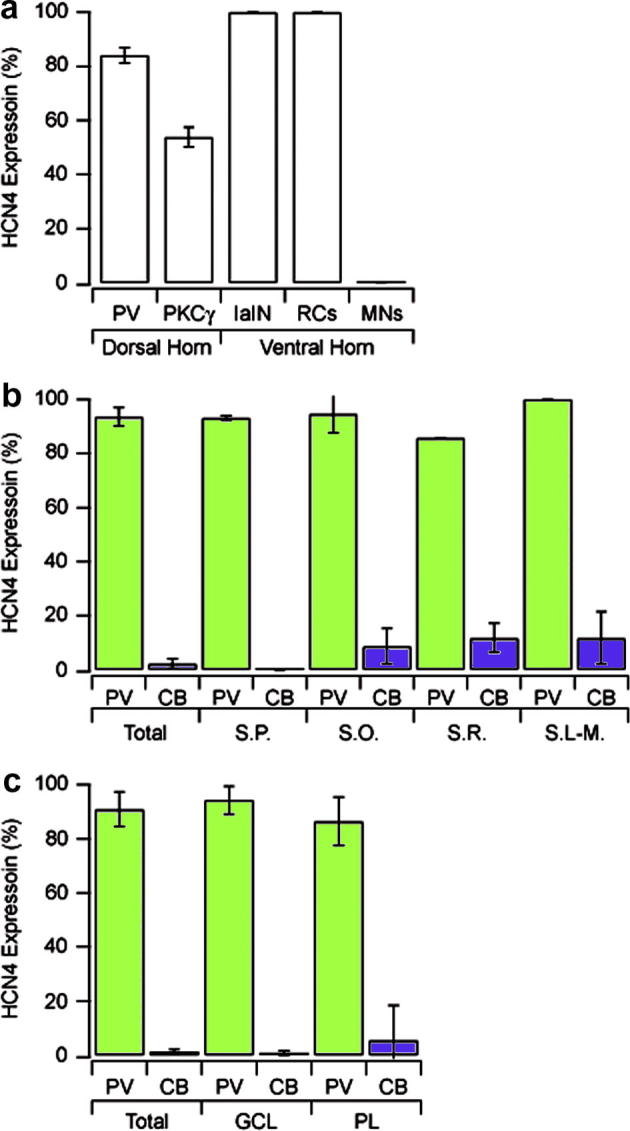
HCN4 expression patterns in the spinal cord, CA1 and dentate gyrus. Relative proportion of HCN4 expression in neurochemically defined populations of neurons in the spinal cord (a), CA1 subfield of the hippocampus (b), and dentate gyrus (c). In panels b and c, data for cells expressing parvalbumin and calbindin are coloured green and blue, respectively.

HCN4-immunolabelling in the hippocampus is found primarily in the *stratum oriens*, at the border of the *stratum oriens* and alveus, in the *stratum pyramidale* and in the cell body layer of the dentate gyrus (Fig. 4). In CA1, HCN4-immunolabelled cells are most prevalent in the *stratum oriens*, at the border of the *stratum oriens* and alveus and in the *stratum pyramidale* (Fig. 5). HCN4-immunolabeled cells are rarely found in the *stratum radiatum* or *stratum lacunosum-moleculare*. A total of 397 PV-IR cells were analysed from the CA1 subfield, of which 372 (93.5%, ±3.40) were immunopositive for HCN4. More specifically, in the *stratum pyramidale* 190 out of 204 PV-IR cells expressed HCN4 (93.1%; ±0.76), compared to 175 out of 185 (94.6%; ±7.04) in the *stratum oriens* and six out of seven (75%; ±17.62) in the *stratum radiatum*. Only one PV-IR cell was found in the *stratum lacunosum-moleculare*, however this cell also expressed HCN4. CB-expressing cells are found principally in the *stratum pyramidale*, with only occasional cells in other layers. A total of 643 CB-IR cells were analysed from CA1, of which only 15 (2.3%; ±1.81) were immunopositive for HCN4. More specifically, in the *stratum pyramidale* none of the 495 CB-IR cells expressed HCN4, compared to seven out of 80 (8.7%; ±6.58) in the *stratum oriens* and six out of 51 (11.8%; ±5.37) in the *stratum radiatum*. Of 17 CB-IR cells seen in the *stratum lacunosum-moleculare*, two expressed HCN4 (11.8%; ± 9.64). In the dentate gyrus, a total of 219 PV-IR cells were analysed from the granule cell and polymorphic layer, of which 199 (90.9%; ±6.38) were immunopositive for HCN4 (Fig. 6). More specifically, 116 of 123 PV-IR cells in the granule cell layer (94.3%; ±5.18) and 83 of 96 PV-IR cells in the polymorphic layer (86.5%; ±8.92) were immunopositive for HCN4. A total of 350 CB-IR cells were also analysed from the granule cell and polymorphic layers, of which five (1.4%; ±0.65) were immunopositive for HCN4. More specifically, only three of 314 CB-IR cells in the granule cell layer (0.9%; ±0.81) and two of 36 CB-IR cells in the polymorphic layer (5.5%; ±12.82) were immunopositive for HCN4. These results for cell populations in CA1 and the dentate gyrus are summarised in Fig. 7b, c respectively.

## Discussion

This study details the pattern of HCN4-expression in neurochemically defined populations of interneurons in the lumbar spinal cord and hippocampus. Our findings suggest HCN4-immunolabelling reliably identifies inhibitory interneurons that exhibit both high-frequency AP discharge and express the *I_h_* sub-threshold current. We also show that adopting the TSA step to amplify HCN4-labelling in our immunocytochemical protocol enhances our capacity to resolve HCN4 expression. This step thus provides an improved and reliable method for sampling large numbers of neurochemically defined interneuron populations with specific physiological properties in the spinal cord and hippocampus.

### Technical considerations

Although a number of studies have used immunocytochemical approaches to examine the distribution of HCN channel subunits in the spinal cord and brain (Antal et al., 2004; Milligan et al., 2006; Brewster et al., 2007), we have modified these protocols and used a TSA step to enhance immunolabelling (Hughes et al., 2012) and demonstrate that this provides improved representation of HCN4 expression in these tissues. We also used short post-fixation times (2 h), because longer times in fixative diminished immunolabelling for HCN channels (DIH, unpublished observations). We have recently used these techniques, in conjunction with targeted whole-cell patch clamp recording experiments, to assess the physiological properties of PV-expressing cells in the mouse spinal dorsal horn (Hughes et al., 2012) and suggested similar approaches could be used to characterise neuronal populations in other transgenic lines. In order to study HCN4 expression in motor neurons, we used a two-step immunocytochemistry protocol using TSA to detect labelling of primary antibodies raised in the same host species (mouse anti-HCN4 and mouse anti-gephyrin) as described previously (Tiong et al., 2011). We found no evidence of double-labelled structures (Fig. 3c insets), thus confirming the selectivity of labelling for either marker using this protocol.

### Neurochemically defined neuronal populations and HCN subunit expression

We have assessed the expression of HCN4 channels in sub-populations of spinal and hippocampal neurons with well characterised electrophysiological, anatomical and neurochemical properties, however other (as yet unidentified) populations of neurons also express HCNs in these regions. The selected populations have been implicated in specific functional roles within discrete neuronal circuits and, in light of recent reports implicating HCN channelopathies in neurological diseases such as epilepsy and pathological pain (Chaplan et al., 2003; Raouf et al., 2010; Lewis and Chetkovich, 2011; Reid et al., 2012), warrant further investigation to determine the potential influence of HCN channel expression (and activity) on sensory processing, modulation of locomotor and hippocampal functions.

Although the terminology used to describe neuronal firing patterns in several regions of the CNS is often similar (e.g. fast spiking), the actual rates of AP discharge within physiologically defined groups from different anatomical areas vary considerably as illustrated in Table 2. For example, spinal interneurons firing at a frequency of 100 Hz are considered tonic firing or fast-spiking cells (Grudt and Perl, 2002; Yasaka et al., 2007; Hughes et al., 2012), however, spinal motor neurons fire at similar frequencies yet are usually described as regular spiking (Button et al., 2008). In contrast, regular-spiking pyramidal and dentate granule cells in the hippocampus typically fire much slower at 3 and 16 Hz respectively (Hájos et al., 2004; Dietrich et al., 2005). Some of these differences are no doubt due to the intrinsic properties of the populations studied. However, variability in experimental protocols (e.g. different membrane holding potentials to record firing patterns) makes it difficult to compare discharge patterns and frequency across studies. It is therefore clear that classifying neurons into functionally distinct groups based on the frequency of their AP discharge patterns is only applicable within given circuits. In this study, the firing patterns we have allocated to each neurochemically defined cell population are based on the most prevalent firing pattern reported for each particular group.

In the spinal dorsal horn, combined electrophysiological and neuroanatomical studies have previously described relationships between morphology, neurotransmitter phenotype and firing patterns in dorsal horn neurons (Grudt and Perl, 2002; Yasaka et al., 2007, 2010). Excitatory interneurons often show delayed, gap or reluctant AP firing patterns, a high incidence of *I_A_* currents and have radial, vertical or central cell-like morphology (Yasaka et al., 2010). In contrast, inhibitory interneurons tend to exhibit tonic- or burst-firing properties, express *I_h_* currents and include islet cells, central cells and vertical cells. The majority (75%) of PV-IR cells in this region are known to express both GABA and glycine (Laing et al., 1994), have islet cell-like morphology (Antal et al., 1990) and are therefore likely to exhibit tonic firing patterns and express *I_h_* currents (Yasaka et al., 2007, 2010). We have recently published data to support this relationship in transgenic mice expressing enhanced green fluorescent protein in PV-positive interneurons in laminae IIi–III (Hughes et al., 2012). Similarly, in the ventral horn the expression of HCN4 in all neurochemically defined IaINs and RCs matches the physiological phenotype of these populations (Renshaw, 1946; Eccles et al., 1954; Mentis et al., 2005; Geertsen et al., 2011). Interestingly, HCN4 was absent in motor neurons even though these cells are known to express *I_h_* currents (Araki et al., 1962; Ito and Oshima, 1965), suggesting that other HCN channel subtypes carry the *I_h_* current in motor neurons. These differences in HCN4 expression are likely to reflect functional disparity in the various physiological properties of these channels between specific populations of cells, further highlighting the need to correlate channel composition and stoichiometry in individual cell types (Graham et al., 2011).

HCN4 expression also appeared to be a faithful marker of fast-spiking interneurons in both the CA1 and dentate gyrus of the hippocampus. Virtually all PV-expressing cells in the CA1 and dentate gyrus express GABA or its synthetic enzyme glutamic acid decarboxylase (Kosaka et al., 1987). Most of these exhibit high-frequency AP discharge patterns (fast-spiking or burst firing patterns) and have axo-axonic or basket cell-like morphology (Pawelzik et al., 2002). For example, PV is expressed in many cells in the *stratum oriens* and at the *stratum oriens-alveus* border and these also tend to have *I_h_* currents and fast-spiking discharge patterns (Maccaferri and McBain, 1996; Tukker et al., 2007). In the dentate gyrus, five types of basket cells have been described (Ribak and Seress, 1983), all of which are thought to express PV and show fast-spiking discharge patterns (Meyer et al., 2002; Aponte et al., 2006). In contrast, the majority of CB-positive neurons are likely to be dentate granule cells with regular-spiking discharge patterns (Maglóczky et al., 1997; Dietrich et al., 2005). Our findings that 90.9% and 93.5% of PV-IR cells in the dentate gyrus and CA1, respectively also express HCN4 reflect the high incidence of *I_h_* currents and fast-spiking discharge patterns in basket cells in these two regions (Koh et al., 1995; Pawelzik et al., 2002).

### Sub-cellular distribution of HCN4 immunolabelling

HCN4-expression in all immunolabelled cells is detectable in both the cell membrane and the cytoplasm. The cytoplasmic expression of HCN4-immunolabelling in hippocampal neurons is stronger than that observed in spinal interneurons, however the significance of this observation is unclear. HCN4 expression in the cell membrane also differs between cells and is likely to reflect subtle differences in the stoichiometry of channels in each subpopulation. For example, the intensity of HCN4-immunolabelling in the soma of PV-expressing cells in the spinal dorsal horn varies (Fig. 2), whereas labelling in RCs and IaINs is typically very strong (Fig. 3a, b). While these observations imply HCN4 is prominent in RCs and IaINs, they also suggest that PV-expressing dorsal horn neurons contain heteromeric HCN channels with moderate amounts of HCN4, or that homomeric HCN4 complexes in these cells are rare. The punctuate nature of HCN4-labelling we report in the membranes of all immunopositive cells is qualitatively similar to expression patterns reported for both HCN1 and HCN2 in other neuronal populations (Luján et al., 2005; Milligan et al., 2006; Hughes et al., 2012). Furthermore, the clear spatial distinction between HCN4- and gephyrin-immunolabelling in somatic and dendritic regions (Fig. 3d) suggests HCN4 channels are only found at extrasynaptic sites, as previously reported for HCN1 channels in the cerebellar cortex, hippocampus, inferior olive and solitary tract nucleus neurons (Notomi and Shigemoto, 2004; Luján et al., 2005; Milligan et al., 2006). The functional implications of this distinct distribution in processing of synaptic inputs and generation of AP outputs remain to be determined but may hint at the importance of HCN4 channels in dendritic integration in these cells.

### HCN channel expression and firing patterns

The four known HCN channel subunits display distinct biophysical properties, are activated at different membrane potentials and have different channel activation kinetics. These factors contribute to the variability of *I_h_* currents in specific cell populations (Wahl-Schott and Biel, 2009; Lewis and Chetkovich, 2011). The expression of each subunit, the stoichiometry of individual channels and the relative distribution of these channels on their somato-dendritic trees are all likely to influence their role in neuron function. Our results show that for the neuronal populations we have studied, there is a strong correlation between HCN4 expression and high-frequency AP discharge, but not necessarily with the expression of *I_h_* currents. Although *I_h_* currents have been observed in both motor neurons and CA1 pyramidal cells (Takahashi, 1990; Magee, 1998, 2000), our findings suggest that these are likely to be mediated through HCN channels with faster kinetics because they lack HCN4 immunolabelling. It is therefore tempting to speculate that the subunit composition of HCN channels in different cell compartments is not uniform, but likely to vary according to the role they serve and the cell population they are expressed in. For example, our results suggest that HCN4 subunit expression in islet cells, RCs, IaINs and hippocampal basket cells could be an important determinant of high-frequency AP discharge, as *I_h_* currents have been shown to influence both spike-firing probability and AP discharge (Southan et al., 2000; Elinder et al., 2006; Dudman and Nolan, 2009; Oswald et al., 2009). Furthermore, excitatory interneurons in the spinal dorsal horn lack *I_h_* currents (Yasaka et al., 2010), however we found approximately 50% of PKCγ cells in lamina II expressed HCN4. This could indicate that HCN channel complexes are important in establishing high-frequency firing patterns in subpopulations of PKCγ cells as these are known to show heterogenous discharge properties (Hu and Gereau, 2011). Regarding the present dataset, both HCN1 and HCN4 are found in PV-expressing dorsal horn interneurons, which typically show high-frequency AP discharge patterns (Hughes et al., 2012). In contrast, HCN1 and HCN2 are expressed in peptidergic C-fibres, which are known to discharge APs at very low frequencies (Antal et al., 2004; Cho et al., 2009; Hachisuka et al., 2010). To further implicate these channels in establishing AP firing patterns, it will be necessary to confirm the presence of HCN channel subunits in the axon initial segment (AIS) of identified neurons, however the experimental design we adopted in this study does not allow us to resolve HCN subunit expression faithfully in these sub-cellular domains. This also serves to highlight the importance of various HCN channel subunit combinations for determining function in specific neuronal populations. Despite attempts to visualise HCN3-immunolabelling in the spinal cord and hippocampal sections, we failed to detect labelling using either a mouse monoclonal or rabbit polyclonal antibody directed against this epitope (data not shown). Although it is possible that the antibodies we used did not detect HCN3 immunolabelling (rabbit anti HCN3 Cat. Number APC-057 from Alomone labs and mouse anti-HCN3, clone N141/28 from NeuroMab), previous studies have suggested that HCN3 expression in these regions of the central nervous system is very low. Thus, we believe HCN3 subunits are unlikely to contribute significantly to establishing and maintaining *I_h_* in these regions (Moosmang et al., 1999; Santoro et al., 2000; Notomi and Shigemoto, 2004).

### HCN channelopathies: implications for pain and other neurological disease

HCN channels have been implicated in both acute and chronic pain as well as a number of neurological diseases (Papp et al., 2006; Chan et al., 2011; Lewis and Chetkovich, 2011). We have previously shown that PV-expressing cells in the mouse dorsal horn are a likely source of presynaptic inhibition to myelinated primary afferent fibres, and that most of these interneurons express *I_h_* currents, exhibit fast-spiking AP discharge and express both HCN1 and HCN4 subunits (Hughes et al., 2012). Although this work did not directly implicate PV-expressing cells in the development of tactile allodynia, a reduction of presynaptic inhibition has been proposed as a mechanism for the development of central sensitisation and tactile allodynia (Wall and Devor, 1981; Laird and Bennett, 1992). As changes in the expression patterns or properties of HCN channels are known to alter AP discharge properties (Southan et al., 2000; Elinder et al., 2006; Dudman and Nolan, 2009), it remains to be seen whether changes in HCN expression in these cells correlate with a reduction in primary afferent depolarisation and the development of allodynia. A number of other channelopathies have been described that alter pain perception and lead to pathological pain (Raouf et al., 2010), while HCN channelopathies have also been implicated in Parkinson’s disease and the development of various forms of epilepsy (Chan et al., 2011; Reid et al., 2012). Given this emerging evidence, an understanding of both the patterns of HCN channel expression in neuronal populations and the roles these channels play in discrete neuronal circuits is essential if we are to establish whether HCN channelopathies can be targeted for the treatment of pain and other debilitating neurological conditions (Maher et al., 2009).

## Figures and Tables

**Table 1:** Primary antibodies

Antigen	Host	Dilution	Antigen	Cat. No.	Source
Calbindin	Rabbit polyclonal	1:1000	Recombinant rat calbindin D-28k	CB-38a	SWANT, Bellinzona, Switzerland. Airaksinen et al. (1997)
ChAT	Goat polyclonal	1:100	Strep-Tag fusion protein of the C-terminal part of rat VAChT (aa 475–530)	139–103	Synaptic Systems, Goettingen, Germany. Alvarez et al. (2005)
Gephyrin (7a)	Mouse monoclonal	1:1000	N-terminus of purified rat gephyrin	147 011/147 021	Synaptic Systems, Goettingen, Germany. Schneider Gasser et al. (2006)
HCN4	Mouse monoclonal	1:500	Fusion protein amino acids 1019–1108 (cytoplasmic C-terminus) of rat HCN4	#75–150 Clone N114/10	UC Davis/NIH NeuroMab Facility, Davis, CA 95616-8519, USA. Giesbrecht et al. (2010) and Khurana et al. (2012)
Parvalbumin	Goat polyclonal	1:500	Rat muscle PV	PVG-214	SWANT, Bellinzona, Switzerland. Schwaller et al. (1999)
PKCγ	Rabbit polyclonal	1:1000	Peptide mapping the C-terminus of mouse PKCγ	C-19	Santa Cruz Biotechnology Inc., Dallas, Texas, USA. Malmberg et al. (1997)

**Table 2:** Properties of neurochemically defined neurons in the spinal cord and hippocampus

Cell type	Neurochemical phenotype	Location	Predominant transmitter phenotype	AP discharge pattern	Discharge frequency (Hz)⁎	HCN4 expression% (# of cells)	References (species)
Dorsal horn INs	PV-IR	Laminae IIi–III	Inhibitory	Tonic/phasic	125 Hz (mean)	84 (*n* = 207)	Hughes et al. (2012) (mouse)

Dorsal horn INs	PKC-γ	Lamina IIi	Excitatory	Delayed	15 Hz (mean)	53 (*n* = 291)	Hu and Gereau (2011) (mouse)
				Tonic/phasic	<30 Hz (mean)		

Renshaw cells	PV-IR, CB-IR	Laminae VII and IX	Inhibitory	Phasic	102 Hz (mean)	100 (*n* = 43)	Mentis et al. (2005) (neonatal mouse)

Ia inhibitory INs	PV-IR, CB-IR	Laminae VII	Inhibitory	Tonic	300 Hz (peak)	100 (*n* = 63)	Geertsen et al. (2011) (cat)

Spinal motoneurons	ChAT	Laminae IX	Excitatory	Tonic	104 Hz (max)	0 (*n* = 102)	Button et al. (2008) (rat)

CA1 INs	PV-IR	*s. pyramidale* (*basket and axo-xonic*)	Inhibitory	Tonic	>100 Hz (mean)	93% (*n* = 204)	Kawaguchi et al. (1987) (rat)
							Pawelzik et al. (2002) (rat)
							Buhl et al. (1994) (rat)
		*s. oriens* (*OLM cells*)	Inhibitory	Tonic	>100 Hz (mean)	95% (*n* = 185)	Maccaferri and McBain (1996) (rat)

CA1 neurons	CB-IR	*s. pyramidale* (*pyramidal cells*)	Excitatory	Regular	3 Hz (mean)	0 (*n* = 495)	Hájos et al. (2004) (rat)
		*s. pyramidale* (*bistratified cells*)	Inhibitory	Regular	>300 Hz (max)		Sík et al. (1995) (rat)

Dentate gyrus	PV-IR	*Granule cell layer*	Inhibitory	Tonic	>100 Hz (max)	94 (*n* = 123)	Meyer et al. (2002) (mouse)
		*Polymorphic layer*				87 (*n* = 96)	Aponte et al. (2006) (rat)

Dentate gyrus	CB-IR	*Granule cell layer*	Excitatory	Regular	16 Hz (mean)	1 (*n* = 314)	Dietrich et al. (2005) (rat)

⁎ Discharge rates were obtained from text/tables in the first instance, or calculated from figures in the listed references.
